# Acute left knee prosthetic joint infection by *Francisella tularensis* with literature review

**DOI:** 10.1016/j.idcr.2023.e01812

**Published:** 2023-06-07

**Authors:** Javier Escovar, Sachin M. Patil, William Roland

**Affiliations:** a Department of Medicine, Division of Infectious Diseases, Clinical Fellow, University of Missouri Hospital and Clinic, 1 Hospital Dr, Columbia, MO 65212, USA; b Department of Medicine, Division of Pulmonary, Critical Care and Environmental Medicine, University of Missouri Hospital and Clinic, 1 Hospital Dr, Columbia, MO 65212, USA; c Department of Medicine, Division of Infectious Diseases, University of Missouri Hospital and Clinic, 1 Hospital Dr, Columbia, MO 65212, USA

**Keywords:** *Francisella tularensis*, Tularemia, Prosthetic joint infection, Doxycycline

## Abstract

Tularemia is a severe zoonotic disease caused by gram-negative bacillus *Francisella tularensis*. *F. tularensis* species account for most cases in the United States of America (USA). Apart from the six classical clinical presentations that include glandular, ulceroglandular, oculoglandular, pharyngeal, typhoidal, and pneumonic, skeletal disease is uncommon. Rare clinical manifestations include primary and secondary skin rashes, erythema nodosum, and erythema multiforme. Infrequent skeletal manifestations have presented as osteomyelitis and prosthetic joint infections. Prosthetic joint infection by *F. tularensis* is a rarity. PubMed literature review revealed a total of five prosthetic joint infection cases. Here we report the sixth and the third case in the USA in a 73-year-old white male with an acute left knee prosthetic joint infection (occurring after a recent episode of left lower extremity cellulitis with septic shock) successfully treated with 14 days of doxycycline.

## Introduction

Prosthetic joint infection (PJI) depends on the patient's multifactorial exposure in daily life. The risk factors and the etiological agents vary from an early PJI to a late PJI. The overall incidence of PJI in total knee arthroplasty (TKA) ten years after surgery is 1.4 % [1]. PJI is the leading cause of TKA failure due to increased stress from weight-bearing and minimal structural support [2]. Native and prosthetic joint infections by Tularemia are highly uncommon. We report a case of *F. tularensis* Left TKA PJI after a recent left lower extremity (LLE) cellulitis episode with septic shock.

## Case presentation

A 73-year-old male with a medical history significant for hypertension, hyperlipidemia, benign prostate hypertrophy, gastroesophageal reflux disorder, depression, sarcomatoid lung cancer on pembrolizumab, pulmonary embolism on rivaroxaban, iron deficiency anemia, bilateral shoulder osteoarthritis (on intraarticular corticosteroid injections every three months), left TKA 2005 presented to his primary care physician for acute left knee worsening painful swelling with no systemic symptoms for last four weeks. The patient denied recent gardening, camping, insect bite, or trauma to the left knee or LLE. The patient had a recent hospitalization five weeks prior for acute LLE cellulitis with septic shock, acute toxic encephalopathy, acute kidney injury treated with intravenous fluids, broad-spectrum antibiotics vancomycin, and piperacillin-tazobactam. The patient had a large circular area of violaceous erythema with warmth above the left ankle joint, with pustules and blisters on the anterior aspect of the shin. Blood cultures were negative. LLE cellulitis improved with antibiotics, and he was discharged on a 10-day course of oral clindamycin. Left knee pain started after antibiotic completion and was initially of low-grade intensity that gradually worsened. The left knee pain was located posteriorly, grade 8/10, worse with flexion and weight-bearing. The pain improved after walking six to seven steps. Vitals were stable and physical examination was benign with elevated inflammatory markers (Table 1a) and a normal left knee x-ray.

**Table 1a:** Laboratory results at Primary care physicians' office.

1) White cell count (3600 – 11,200/mL)	5900
2) Hemoglobin (13.1 – 16.8 gm/dL)	9.6
3) Hematocrit (38.2–48.4 %)	30.3 %
4) Platelet count (150,000 – 400,000/mL)	458,000
5) Uric acid level (3.5 – 7.2 mg/dL)	3.4
6) C-Reactive protein (0 – 0.5 mg/dL)	7.39
7) Erythrocyte sedimentation rate (0 – 20 mm/hr)	82

**Fig. 1:**
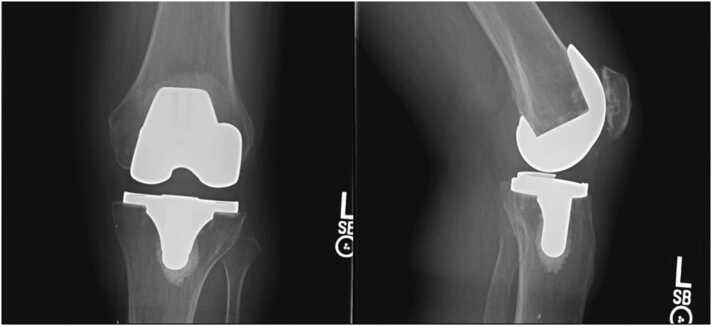
Anteroposterior and lateral view of left knee (at Orthopedic clinic) revealed primary cemented components with good alignment and no evidence of loosening.

He was then referred to an orthopedic clinic for a left knee PJI concern, where examination revealed a non-erythematous left knee incision line without drainage but mild effusion and left hamstring muscle pain. Left knee range of motion was 0–90 degrees, and the sensation was intact to light touch. Left knee x-ray, two views revealed primary cemented components with good alignment and no evidence of loosening (Fig. 1). Left knee joint aspiration revealed 16 mL of clear straw-colored synovial fluid. Synovial fluid analysis (Table 1b) indicated acute infection, and culture revealed *F.tularensis* on the Vitek-2 system. The infectious disease team started him on doxycycline 100 mg twice daily for two weeks. The specimen was then subcultured on modified Thayer-Martin agar media for confirmation (laboratory personnel were notified), and a sample was sent to the state laboratory for confirmation. The state laboratory confirmed *F. tularensis* growth, and a convalescent antibody titer two weeks later was high at 1:640. After the antimicrobial therapy completion, his symptoms had subsided at the primary care follow-up. He had no pain with the left knee range of motion and weight-bearing. He has been symptom-free for the last twelve months and follows up with his primary care physician.

**Table 1b:** Left knee synovial fluid analysis at orthopedic clinic.

1) Total cells per cubic millimeter	9299
2) Total White blood cells per cubic millimeter	3299
3) Total Red blood cells per cubic millimeter	6000
4) Neutrophils	41 %
5) Lymphocytes	46 %
6) Others	15 %

## Discussion

Tularemia is an uncommon zoonosis with an incidence of 0.05 per 100,000 population in the USA caused by *F. tularensis* [3]. Multiple strains exist with variable virulence and severeness of clinical presentation, often during spring to early fall. Transmission vectors include ticks and deer fly in contact with an infected cottontail rabbit or a muskrat. The ticks frequently involved are the lone star (*Amblyomma americanum*), wood (*Dermacentor andersoni*), and dog tick (*Dermacentor variabilis*) in the USA [3]. None of the six classical presentations involve the skeletal system, which is rare and a complication. Cellular immunity develops within a few weeks and confers potent lifelong protection after infection in a healthy patient [3]. Recurrent infections are rare, with an ulceroglandular presentation and no systemic symptoms [3]. PJI occurs via hematogenous seeding from tularemia, inhalation of contaminated aerosols directly via tick bite, and lymphohematogenous spread. Hematogenous seeding is frequent in prosthetic joints due to the smaller inoculum needed, and prosthesis-induced acquired local granulocyte defect [4]. *F. tularensis* virulent attributes include type IV pili (adherence), polysaccharide capsule (inhibits IgM and C3 binding), capsule-like complex, or high molecular weight carbohydrate (stops antibody binding and complement deposition) [3], [5], and acid phosphatases (aid survival in macrophages). Biofilm formation does help its survival in the environment, and its role in PJI is unclear [5]. Except for a single case of osteomyelitis due to a cat bite, no native joint infections have been reported [6]. *F. tularensis* probably lacks synovial tropism due to a lack of fibronectin-binding ability or adhesins.

PubMed literature review revealed 5 cases of *F. tularensis* PJI (Table 2) [7], [8], [9], [10]. Including our patient, two were from Europe, and four were from North America. All patients were over 50 years old at presentation, predominantly males, with the knee joint being commonly affected. The frequent infection source was suspected tick bite to a lower extremity. Two patients underwent surgical revision, and the rest were treated with antimicrobials plus diagnostic or therapeutic aspiration. Two patients were on immunosuppression [7]. *F. tularensis* was isolated in culture from the synovial fluid in five patients and periprosthetic tissue in one patient. Serological assays were done in four patients, and 16 S rRNA (ribosomal Ribonucleic acid) analysis in three patients. The recommended antimicrobials are streptomycin/gentamicin, doxycycline, or quinolones, and the duration is unclear for PJI [3]. Currently, no guidelines exist to address *F. tularensis* PJI. It is unclear whether a dual antimicrobial combination is better than a single agent. One patient showed an excellent response to a combination of ciprofloxacin and rifampin than ciprofloxacin alone, possibly due to *F. tularensis* biovar type B PJI [7]. The fact that only one patient is on chronic antimicrobial suppressive therapy (delayed diagnosis and treatment) than others treated for a specific duration may suggest a lack of or ineffective biofilm formation, protective effect of cellular immunity, or a timely detection and treatment with a better response [10]. Unfortunately, no serological test was done to confirm acquired immunity in this patient. A fraction of culture-negative PJI due to *F. tularensis* could have been treated inadvertently with empirical regimens, including quinolones. Suppressive therapy may have a role if symptoms persist.

**Table 2:** Patient characteristics in Prosthetic joint infections (PJI) by *F. tularensis*[8].

Reference	1) Cooper CL [7]	2) Chrdle A [8]	3) Chrdle A [8]	4) Rawal H, [9]	5) Azua EN [10]	6) Current case
Country	Ontario, Canada	Switzerland	Switzerland	Illinois, USA	Colorado, USA	Missouri, USA
Age/Gender	68/Male	84/Female	84/Male	77/Male	58/Male	73/Male
Infection source	Hunter (Tick bite six months prior to surgery)	Suspected rabbit dust inhalation	Suspected typhoidal tularemia	Hunter (suspected animal exposure)	Farmer (Suspected rabbit carcass exposure)	Suspected tick bite six weeks before
Immunosuppression	Methotrexate	None	None	None	None	Pembrolizumab, Intra-articular shoulder Corticosteroids
Diagnosis Year	1998	2016	2016	2017	2020	2021
Joint involved	Knee	Knee	Knee	Hip	Knee	Knee
Time interval to diagnosis after last surgery	1 year	12 years	8 years	1 week after revision of total hip arthroplasty (25 yrs)	5 months after the revision	16 years
Clinical Features	Painful swelling and copious incision line serous drainage with no systemic symptoms.	Joint redness and pain with no systemic symptoms.	Abdominal pain, encephalopathy, painful swelling with fever.	Right hip pain and fever.	Bilateral joint recurrent effusion with no systemic symptoms.	Painful joint swelling with effusion and no systemic symptoms.
Inflammatory markers	ESR 47 mm/hr CRP not done	ESR 69 mm/hr CRP 8.1 mg/dL	ESR not done CRP 9.8 mg/dL	ESR 96 mm/hr CRP 16.20 mg/dL	ESR not done CRP not done	ESR 82 mm/hr CRP 7.39 mg/dL
Diagnostic method	Culture (synovial fluid)	Culture and 16 S rRNA analysis (tissue culture)	Culture and 16 S rRNA analysis (synovial fluid)	Culture and MALDI-TOF MS (synovial fluid)	Culture and 16 S rRNA analysis (synovial fluid)	Vitek-2 and culture (synovial fluid)
Serology	Antibody titer of 1:320	IgM 232.6 (<10 U/mL) IgG 126.4 (< 0 U/mL)	Antibody titer of 1:80	Not done	Not done	Antibody titer of 1:640
Surgical treatment	2 stage revision	2 stage revision	Therapeutic aspiration with retention	Retention after diagnostic aspiration	Therapeutic aspiration with retention	Retention after diagnostic aspiration
Antimicrobial Choice/duration	Ciprofloxacin and Rifampin for five to six months.	Doxycycline for six weeks	Doxycycline (20 days) and Gentamicin (10 days), followed by 20 days of ciprofloxacin	Doxycycline for one year	Doxycycline Chronic suppression	Doxycycline for 14 days

ESR = Erythrocyte sedimentation rate, CRP = C-Reactive protein, RNA = Ribonucleic acid,

MALDI-TOF MS = matrix-assisted laser desorption ionization-time of flight mass spectrometry.

Our patient resides in a Mid-Missouri county rural area farmhouse with no pets or animal exposure. He denied recent visits to parks or consuming contaminated agricultural products and water. Our patient presented after a suspected ulceroglandular lesion from a tick bite with a superimposed bacterial infection and septic shock. His symptoms started immediately after the completion of the clindamycin course. Clindamycin demonstrates an antibiofilm effect, excellent bone, and joint penetration, and is bactericidal against *F. tularensis* [11], [12], [13]. Our patient had an acute PJI, and symptoms resolved after arthrocentesis and antimicrobial therapy [14]. Clindamycin inadvertently might have contributed in treating our patient's acute PJI. Despite being on pembrolizumab and intraarticular steroids, he has remained symptom-free currently. It is unknown if the acquired robust immunity after *F. tularensis* infection has any protective role in preventing PJI recurrence or relapse.

## Conclusion

Due to advances in joint prostheses, THA and TKA are being done in a younger population compared to a few decades back. The incidence of *F. tularensis* PJI might increase over the next few decades due to better diagnostic processes and activity resumption post-surgery. Obtaining occupational and environmental exposure is imperative before THA/TKA. Due to the lack of definite therapeutic guidelines, the *F. tularensis* PJI treatment approach is individualized based on patient risk factors. A high degree of clinical suspicion for *F. tularensis* infection in culture-negative PJI should be entertained as an earlier diagnosis can help in specific antimicrobial therapy, prevent surgical interventions, and retain the prosthesis.

## Ethics approval

Care was taken to ensure that all patient identifiers were removed in the process of creating this case report, the patient was made aware of this case report.

## Funding

There are no sources of funding to report for this case report.

## CRediT authorship contribution statement

**Javier Escovar** - Principal author. **Sachin M Patil** - Co-author, Faculty advisor and contributor. **William Roland** - Faculty advisor and contributor.

## Declaration of Competing Interest

The authors declare that they have no competing interests.
